# Cytoplasmic Expression of Pontin in Renal Cell Carcinoma Correlates with Tumor Invasion, Metastasis and Patients’ Survival

**DOI:** 10.1371/journal.pone.0118659

**Published:** 2015-03-09

**Authors:** Xiang Zhang, Juchao Ren, Lei Yan, Yueqing Tang, Wenhua Zhang, Dawei Li, Yuanwei Zang, Feng Kong, Zhonghua Xu

**Affiliations:** 1 Department of Urology, Qilu Hospital, Shandong University, Jinan, Shandong Province, China; 2 Institute of Basic Medical Science and Key Laboratory of Cardiovascular Proteomics of Shandong Province, Qilu Hospital, Shandong University, Jinan, Shandong Province, China; 3 Central Laboratory, Shandong University Second Hospital, Shandong University, Jinan, Shandong Province, China; University of Texas Health Science Center, UNITED STATES

## Abstract

Renal cell carcinoma (RCC) is the most lethal of all genitourinary malignancies. Distant metastasis represents the major cause of death in patients with RCC. Recent studies have implicated the AAA+ ATPase pontin in many cellular activities that are highly relevant to carcinogenesis. In this study, we demonstrate for the first time that pontin was up-regulated in RCC, and plays a previously unknown pro-invasive role in the metastatic progression of RCC through epithelial-to-mesenchymal transition (EMT) pathway. 28 pairs of freshly frozen clear cell RCC samples and the matched normal renal tissues analyzed by quantitative RT-PCR and western blotting demonstrated that pontin was up-regulated in clear cell RCC tissues than in normal renal tissues. In addition, immunohistochemistry was used to evaluate subcellular pontin expression in 95 RCC patients, and found that overexpression of pontin in cytoplasm positively correlated with the metastatic features, predicting unfavorable outcomes of RCC patients. Furthermore, *in vitro* experiments show pontin was predominantly expressed in cytoplasm of RCC cell lines, and a significant suppression of cell migration and invasion in pontin siRNA treated RCC cell lines was observed. Mechanistic studies show that pontin depletion up-regulated the E-cadherin protein and down-regulated vimentin protein, and decreased nuclear β-catenin expression, suggesting the involvement of EMT in pontin induced metastatic progression. Together, our data suggest pontin as a potential prognostic biomarker in RCC, and provide new promising therapeutic targets for clinical intervention of kidney cancers.

## Introduction

Renal cell carcinoma (RCC) is the most common malignances of adult kidneys. Due to the asymptomatic nature of early stage RCC, more than one third of the patients present with locally advanced or metastatic disease at the time of diagnosis. And the prognosis of the patients with metastatic RCC is extremely poor with a 5-year survival rate less than 9% [1]. Understanding the molecular mechanisms of RCC metastatic progression will help identify novel potential molecular targets for the development of effective therapy. However, the study on searching for molecular targets in RCC that has clinical significance remains substantially limited.

Pontin and reptin belong to the AAA+ (ATPase associated with diverse cellular activities) superfamily, which are highly evolutionarily conserved. Pontin and reptin are usually co-expressed [2]. Both of them are found in several multi-protein complexes, where they are thought to participate notably in chromatin remodeling [3], transcriptional regulation [4], DNA damage repair [5], and small nucleolar RNA biogenesis [6], but they can also function independently from each other. Notably, they interact with several oncogenic transcriptional factors, such as *β*-catenin and c-myc, and modulate their activities [7–9]. Functional studies indicated both proteins participating in tumorigenic activities. Silencing of pontin *in vitro* led to tumor growth arrest [10], and silencing of reptin in vivo significantly reduced tumor progression within xenografts in mice [11]. These features suggest their potential in being good targets for cancer therapy. Aberrant expression of pontin and/or reptin has been reported in malignancies of liver [10, 12], colon [13, 14], breast [15], among others. However, the expression and biological function of pontin in RCC has so far not been elucidated.

In our previous study, we firstly reported that reptin is overexpressed in RCC, and cytoplasmic expression of reptin predicts an unfavorable outcome for RCC patients [16]. Now we presume that pontin, as a homology partner of reptin, may also play an important role in RCC metastasis. The present study was performed to examine the postulated oncogenic role of pontin in RCC. In this respect, we examined pontin expression in RCC surgical samples, and explored the potential effects of pontin on RCC metastatic progression and patients’ survival. In addition, we applied siRNA to knockdown pontin in RCC cell lines to examine its biological functions and the possible molecular mechanisms.

## Materials and Methods

### Ethics statement

The study was approved by Ethics Boards of Qilu Hospital, Shandong University. All tissue sample acquisition was carried out according to the institutional guidelines, and all subjects signed written informed consent.

### Clinical RCC specimens and RCC cell lines

For immunohistochemical analysis, tissue samples from 95 RCC patients who underwent radical nephrectomy were collected between June 2005 and January 2009, at the Department of Urology, Qilu Hospital, Shandong University, Jinan, China. None of the patients had received preoperative chemotherapy or radiotherapy. For survival analysis, more patients with advanced clinical stage were enrolled in this study. After surgery, 95 tumor samples and 14 normal renal tissues from the tumor bearing kidneys were fixed in formalin, embedded in paraffin and stored in the Department of Pathology, Qilu Hospital. For all samples, staging was reevaluated and determined according to the tumor node metastasis staging system [17], and the nuclear grade was evaluated on the basis of the Fuhrman four-grade scale [18]. All patients had their follow-up in the outpatient clinic of our department until April 2014. Patients without complete follow-up data were excluded from this study. Detailed clinicopathological information is listed in Table 1.

**Table 1:** Characteristics of the patients with RCC and correlations between subcellular pontin expression and clinicopathological variables (n = 95).

Variables	No. of pts	Cytoplasmic pontin expression		*P*-value	Nuclear pontin expression		*P*-value
		High	Low		High	Low	
*Patients' age*							
≥ 55 Years	38	9	29	0.511	9	29	0.229
< 55 Years	57	17	40		8	49	
*Gender*							
Male	75	19	56	0.389	15	60	0.512
Female	20	7	13		2	18	
*T stage*							
T_1_	41	8	33	0.057	9	23	0.351
T_2_	22	4	18		5	17	
T_3_	21	8	13		1	20	
T_4_	11	6	5		2	9	
*N stage*							
N_0_	84	22	62	0.486	15	69	0.979
N_1,2_	11	4	7		2	9	
*M stage*							
M_0_	83	19	64	0.010	13	70	0.218
M_1_	12	7	5		4	8	
*TNM stage*							
I,II	56	10	46	0.003	11	45	0.594
III,IV	39	16	23		6	33	
*Histological grade*							
G_1,2_	47	8	39	0.025	4	43	0.030
G_3,4_	48	18	30		13	35	
*Histological type*							
Clear cell	68	17	51	0.168	12	56	0.849
Papillary	13	6	7		3	10	
Chromophobe	10	1	9		1	9	
Unclassified	4	2	2		1	3	

Abbreviations: RCC = renal cell carcinoma.

For quantitative real-time PCR (qRT-PCR) and Western blotting analysis, 28 pairs of pathologically confirmed clear cell RCC (ccRCC) specimens (the tumor tissues and the matched normal renal tissues from the tumor bearing kidneys) were obtained during radical nephrectomy between July 2013 and October 2013 in our department. After surgery, every sample was immediately stored in liquid nitrogen until use.

Human RCC cell lines A498, 786-O and non-malignant-immortalized renal cell line HK-2 were purchased from ATCC (Manassas, VA, USA), and Human RCC cell line KRC/Y was obtained from the MTC-KI (Stockholm, Sweden) cell line collection [19]. HK-2 cells were cultured in KSFM medium (Gibco, USA), and other cells were cultured in RPMI-1640 medium (Invitrogen, Carlsbad, CA, USA) containing 10% FBS (Invitrogen), 100 U/ml penicillin (Sigma, St. Louis, MO, USA), and 100 μg/ml streptomycin (Sigma). All cells were cultured in an incubator maintained at 37°C, 5% CO_2_.

### Small interfering RNA treatment

The chemical modified siRNA targeting pontin and control siRNA were purchased from Invitrogen. The sequence of siRNA was pontin siRNA1: 5'-CCA UGC UGU AUA CUC CAC AGG AAA U-3' [20], and pontin siRNA2: 5'- TAA AGG AGA CCA AGG AAG T-3' [5]. Cells were transfected with either pontin or control siRNA with concentration of 100 nM using Lipofectamine 2000 (Invitrogen) following the manufacturer’s instructions. The expression efficiencies were measured by quantitative RT-PCR and Western blotting.

### RNA extraction, reverse transcription-PCR and quantitative real-time PCR

Total RNA was extracted from frozen surgical specimens and cell lines transfected for 48 h using Trizol reagent (Invitrogen), and reverse transcribed into cDNA using M-MLV reverse transcriptase (TaKaRa, Biotechnology, China). qRT-PCR was performed in a Mastercycler ep realplex real-time PCR system (Eppendorf, Hamburg, Germany) using SYBR Green kit (Applied Biosystems, Foster City, CA, USA) and the following primers: pontin, 5'-GGCATGTGGCGTCATAGTAGA-3' (Forward) and 5'- CACGGAGTTAGCTCTGTGACT-3' (Reverse), C-myc, 5’-G-3’ (Forward) and 5’-G-3’ (Reverse), Cyclin-D1: 5’-G-3’ (Forward), and 5’-G-3’ (Reverse) and *β*-actin: 5'-AGTTGCGTTACACCCTTTCTTG-3' (Forward) and 5'-CACCTTCACCGTTCCAGTTTT-3' (Reverse). *β*-actin was used as an internal control, and the relative pontin mRNA expression levels were analyzed using the 2^-ΔΔCt^ method.

### Immunohistochemistry

Immunohistochemistry was performed using a two-step protocol as we described previously [16]. The primary antibody used was anti-pontin (1:150; Abcam, Cambridge, MA, USA; Cat. No. ab51500). Color was developed with DAB Horseradish Peroxidase Color Development Kit (Beyotime, Haimen, China; Cat. No. P0202). Then the slides were counterstained with haematoxylin.

### IHC evaluation and selection of the cutoff point

The slides were scored by 2 independent pathologists, who were blind to the patient characteristics. The cytoplasmic and nuclear pontin staining were evaluated separately by a semi-quantitative method considering both staining intensity (0, no staining of the tumor cells; 1, mild staining; 2, moderate staining and 3, strong staining) and the percentage of positive staining (0, 0–5%; 1, 6%-25%; 2, 26%-50%; 3, 51%-75% and 4, >76%). The immunoreactive scores were determined by the sum of intensity and extension. Conflicting scores were resolved by consensus.

Because the immunoreactive scores of pontin showed neither a biphasic distribution nor a clear negative value, we determined the optimal cutoff point based on a heterogeneity value measured by log-rank test as literature reported [21]. Briefly, each staining index score from 1 to 7 was used as cutoff point, at each of these cutoff points, the patients were divided into two groups and the differences in OS were analyzed by log-rank test. The greatest difference between the two groups was at the staining index score 5. Therefore, this score was chosen as cutoff point for discrimination between pontin low and high expression.

### Fluorescent immunocytochemical analysis

Cells were seeded onto coverslips, fixed and permeabilized with ice-cold methanol for 10 min. Then slides were blocked with 5% donkey serum, and incubated with mouse anti-pontin (1:100; Abcam) or mouse anti-*β*-catenin (1:100; BD Biosciences Pharmingen, San Jose, CA) antibodies overnight at 4°C. Afterwards, slides were immunostained with Fluor 488-conjugated donkey anti-mouse antibody for 1 hour at room temperature, followed by nuclear counterstaining with DAPI. Images were acquired using an epifluorescence microscopy (Nikon Eclipse TE 2000-U).

### Western blotting analysis

Proteins extracted from frozen surgical specimens and cells transfected for 72 h were immunoblotted with different antibodies as we described previously [16]. The primary antibodies used were anti-pontin (1:100; Abcam), anti-E-cadherin (1:500; proteintech, Chicago, IL, USA; Cat. No. 20874-1-AP), anti-vimentin (1:1000; proteintech, Cat. No. 10366-1-AP) and anti-*β*-actin (Santa Cruz Biotechnologies, Santa Cruz, CA, USA).

### Wound healing assay

Cells were seeded into 6-well plates the day before siRNA transfection. 24 h after transfection, similar sized wounds were made across the confluent cell monolayer with 200 μl pipette tips. The plates were washed twice to remove the detached cells and then incubated in serum-free medium for 24 h. Digital pictures were taken at 0 and 24 h after the scratch. The migration rate was calculated using the following formula: the radios of wound closure = decreased wound area at 24 h/wound area at 0 h.

### Matrigel invasion assay

Cell invasion assay was performed using a 24-well transwell chamber with a pore size of 8 μm (Costar, NY, USA). The insert of the pore was coated with 50ul Matrigel (dilution at 1:2, BD Bioscience). Cells were trypsinized after transfection for 48 h and 1 × 10^5^ cells were transferred to the upper chamber in 100 μl of serum-free medium. The lower chamber was filled with 1640 containing 10% FBS as chemoattractants. After incubation for 24 h, the non-invaded cells on the upper membrane surface were removed with a cotton swab, and the membranes were fixed and stained using 0.1% crystal violet. Representative fields were digitally captured and the invaded cells were counted in five randomly selected high-power fields (× 400) per filter to assess the average number of invaded cells.

### Statistical analysis

The relative pontin expression level in the matched tumor and normal renal tissues was calculated using paired *t*-test. The correlations between subcellular pontin expression and the clinicopathological variables were analyzed using Chi-square test and Fisher’s exact test. The survival analysis was evaluated using Kaplan-Meier method compared by the log-rank test. Multivariate Cox regression analysis was used to identify independent prognostic factors. The data of independent experiments *in vitro* were expressed as mean ± s.e.m. and analyzed by Student’s *t*-test. All statistical analyses were performed with SPSS 16.0 statistical software (Chicago, IL, USA). *P* value < 0.05 was considered statistically significant.

## Results

### Expression of pontin and E-cadherin in clinical RCC samples

**Fig 1:**
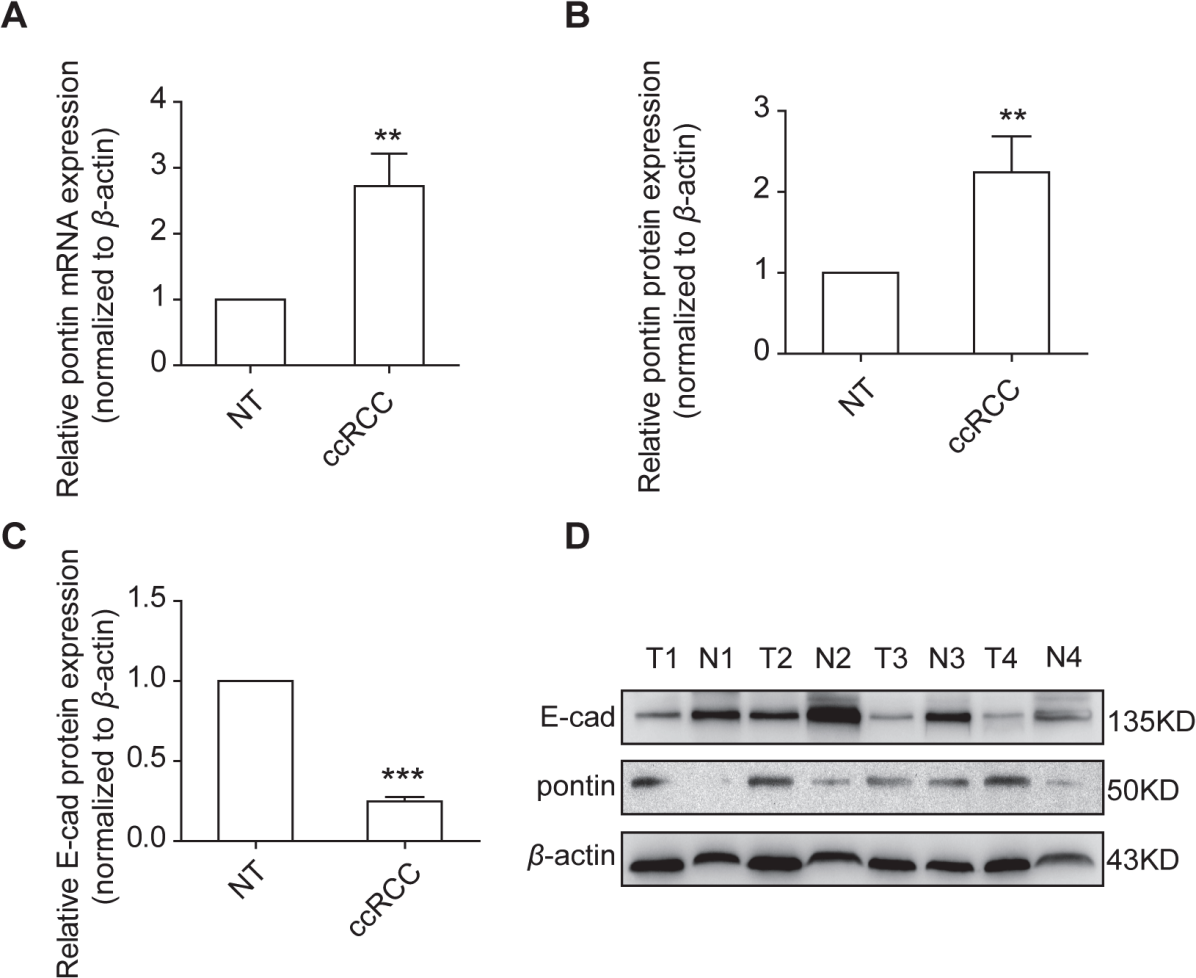
Pontin and E-cadherin expression profiles in 28 pairs of ccRCC tissues and the matched normal renal tissues. (A) Relative pontin mRNA levels determined by qRT-PCR. (B and C) Relative pontin and E-cadherin protein levels determined by western blotting analyses. (D) Western blotting analyses from 4 representative pairs of matched ccRCC and normal renal tissues. *β*-actin was used as a loading control. **P<0.01, ***P<0.001.

Expression of pontin mRNA and protein levels were detected using qRT-PCR and western blotting in 28 paired freshly frozen ccRCC tissues and the matched normal renal tissues. Pontin mRNA was significantly up-regulated in ccRCC tissues than in matched normal renal tissues (*P* = 0.0016, Fig. 1A). Consistent with the pontin mRNA expression profile, pontin protein was also increased in ccRCC tissues (*P* = 0.0095, Fig. 1B and D). Interestingly, we also found a significant decrease of the E-cadherin protein expression in ccRCC tissues, compared to the matched normal renal tissues (*P* < 0.001, Fig. 1C and D).

### Subcellular pontin expression and the clinicopathological variables of RCC patients

**Fig 2:**
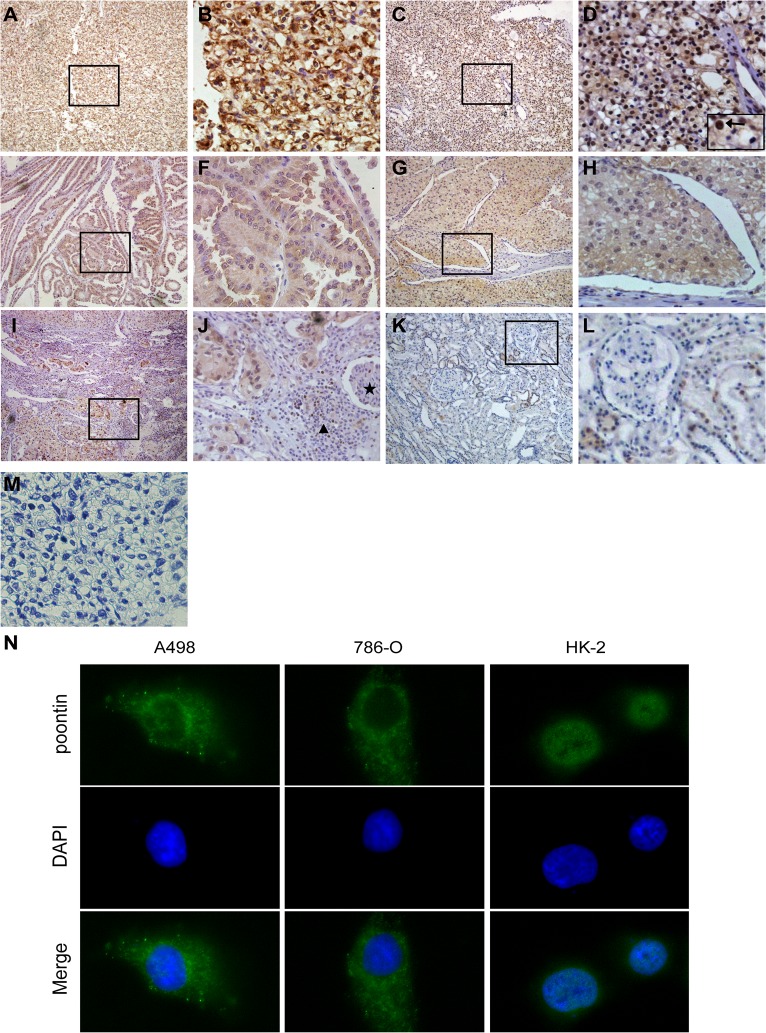
Immuno staining of pontin in RCC surgical specimens and cell lines. Strong cytoplasmic staining of pontin in clear cell RCC (A and B), papillary RCC (E and F), and chromophobe RCC (G and H). Strong nuclear staining of pontin in clear cell RCC (C and D, arrow shows the cells with a strong nuclear staining). Strong cytoplasmic staining of pontin in the tumorous invasive margin of clear cell RCC, while no pontin staining or predominantly nuclear pontin staining in tumor adjacent renal tissues (I and J, ★: glomerulus of the tumor adjacent renal tissues; ▲: infiltrating lymphocytes). Diffuse pontin staining in the proximal tubules of normal renal tissues (K and L). Negative control of clear cell RCC (M). Subcellular localization of pontin protein in RCC cell lines A498, 786-O and normal renal epithelial cell line HK-2 detected by fluorescent immunocytochemical analysis (N). Magnification: A, C, E, G, I, K: ×100; B, D, F, H, J, L, M: ×400 and N: ×600.

We further analyzed pontin protein expressions in a set of 95 RCC surgical specimens and 14 tumor adjacent renal tissues using an immunohistochemical approach. Representative staining of pontin is shown in Fig. 2A-M. The presence of pontin protein was found in 87 of 95 (91.6%) RCC samples, and pontin protein expression was highly expressed in 38 out of the 87 (43.7%) pontin-positive RCC patients, whereas only weak to moderate pontin staining was found in 8 of 14 (57.1%) tumor adjacent renal tissues. In addition, positive staining for pontin was observed both in cytoplasm and in nucleus (Fig. 2A-H). Interestingly, among the 95 RCC specimens there are 5 samples presented with both the tumor tissues and the tumorous invasive margin, and strong cytoplasmic staining of pontin was observed at all of the tumorous invasive margin (Fig. 2I and J). However, only a diffuse pontin staining was observed in the proximal tubules of normal renal tissues, and predominantly localized in nucleus (Fig. 2K and L). In addition, fluorescent immunocytochemical analysis showed pontin expression was predominantly localized in the cytoplasm of RCC cell lines A498 and 786-O, while a weak nuclear pontin expression was observed in the normal renal epithelial cell line HK-2 (Fig. 2N), which was in accord with the IHC observation.

Because of the different IHC staining pattern of pontin in RCC tissues, as compared with the tumor adjacent renal tissues, cytoplasmic and nuclear pontin staining were scored separately, and its significance with respect to the main clinicopathological variables was evaluated (Table 1). Patients with high cytoplasmic pontin expression exhibited a significant association with advanced clinical stage (*P* = 0.003), poor histological grade (*P* = 0.025) and distant metastasis (*P* = 0.010). In contrast, we only found one significant association between high nuclear pontin expression and poor histological grade (*P* = 0.030). Furthermore, no significant associations between pontin expression and patients’ gender, age or histological type was observed.

### Subcellular pontin expression and postoperative survival of RCC patients

**Fig 3:**
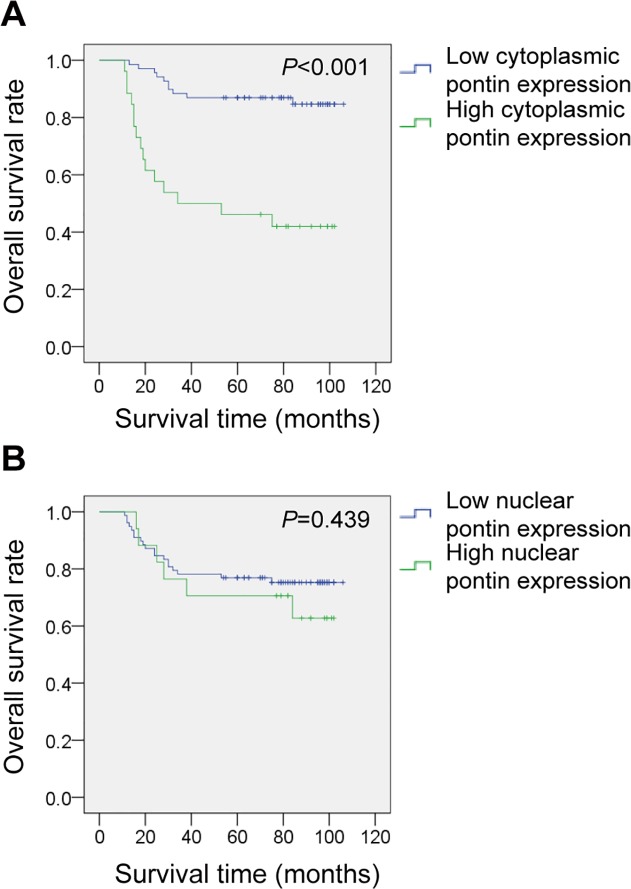
Kaplan-Meier curves of overall survival stratified by cytoplasmic pontin expression status. (A) and nuclear pontin expression status (B). *P*-value was calculated by log-rank test.

Of the total number of patients, 25 (26.3%) patients died of RCC with a median follow-up time of 24 months (11∼84 months), and the remaining 70 (73.7%) patients were alive with a median follow-up time of 92 months (54∼106 months). Kaplan-Meier analysis compared by the log-rank test was used to evaluate the effects of the clinicopathological variables on overall survival (OS). Univariate analysis indicated that patients with advanced primary tumor stage, lymph nodes metastasis, distant metastasis, high histological grade and advanced clinical stage exhibited worse outcomes (all *P* <0.01; Table 2). And a significant lower OS rate in patients with high cytoplasmic pontin expression was observed as compared to those with a low level (85.5% *vs*. 42.3%, *P* < 0.001; Fig. 3A). However, there was no significant difference in terms of OS between high and low nuclear pontin expression (*P* = 0.439; Fig. 3B). In addition, multivariate analysis indicated that advanced clinical stage, high histological grade and high cytoplasmic pontin expression were independent prognostic factors for RCC patients (Table 2).

**Table 2:** Univariate and Multivariate analyses for overall survival of 95 RCC patients.

Variables	Univariate analysis		Multivariate analysis		
	Survival rate	*P*-value	HR	95% CI	*P*-value
Patients' age (≥55 yrs vs < 55 yrs)	77.2% vs 68.4%	0.518			
T stage (T_1,2_ vs T_3,4_)	93.7% vs 34.4%	< 0.001	0.79	0.367–2.720	0.559
N stage (N_0_ vs N_1,2_)	78.6% vs 36.4%	0.002	1.19	0.351–4.048	0.778
M stage (M_0_ vs M_1,2_)	84.3% vs 0	< 0.001	3.65	0.769–17.336	0.103
TNM stage (I, II vs III, IV)	100% vs 35.9%	< 0.001	7.94	2.277–27.708	0.001
Histological grade (G_1,2_ vs G_3,4_)	89.4% vs 58.3%	0.001	2.39	1.194–4.801	0.014
Cytoplasmic pontin expression (Low vs High)	85.5% vs 42.3%	< 0.001	2.67	1.124–6.337	0.026
Nuclear pontin expression (Low vs High)	75.6% vs 64.7%	0.439			

Abbreviations: RCC = renal cell carcinoma; HR = hazard radio; CI = confidence interval.

### Effects of pontin depletion on cell migration and invasion

**Fig 4:**
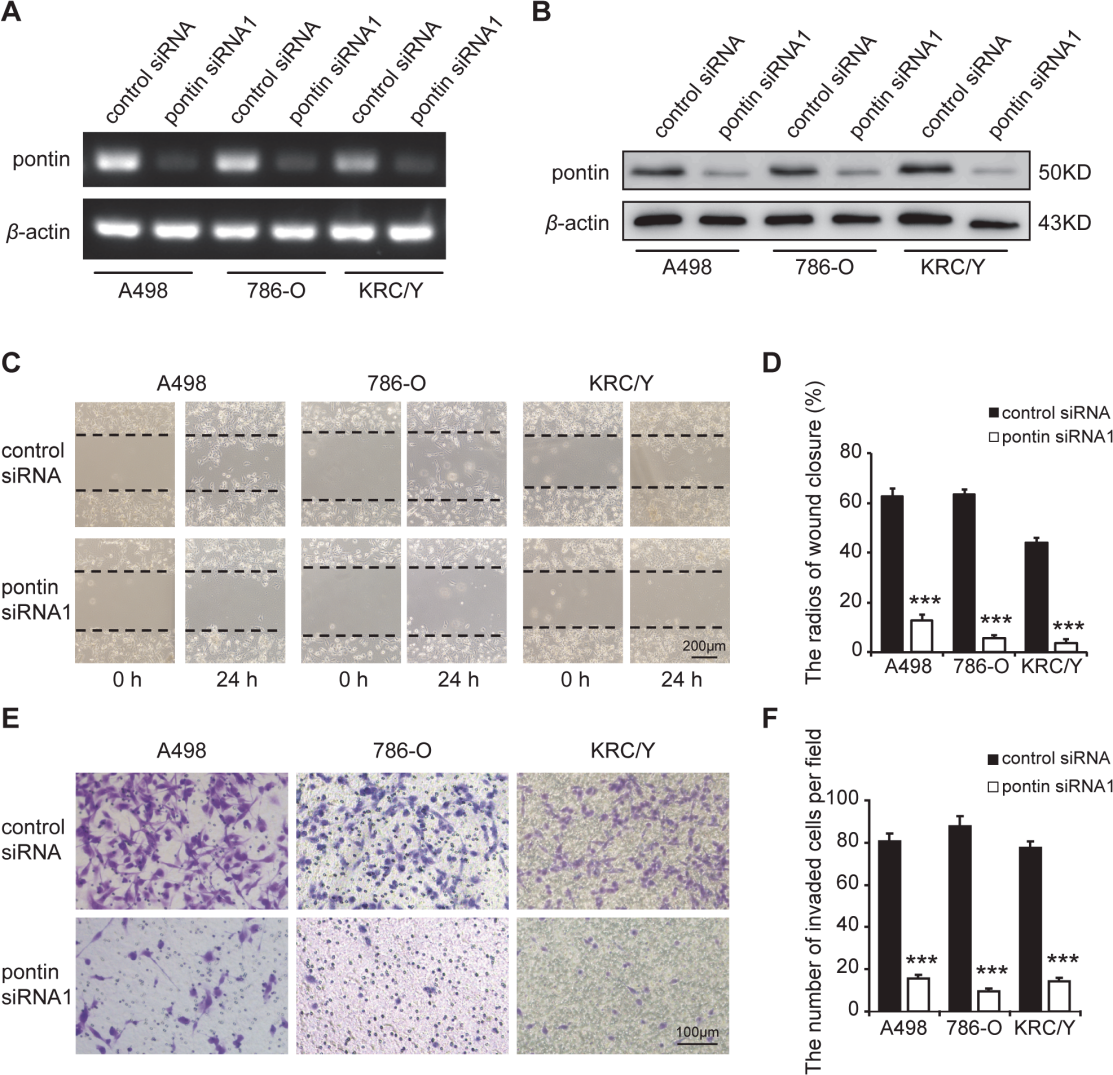
Effects of pontin depletion on RCC cell migration/invasion activities *in vitro*. (A) Effects on the pontin mRNA expression after siRNA transfection for 48 h detected by semi-quantitative RT-PCR. (B) Effects on the pontin protein expression after siRNA transfection for 72 h detected by western blotting. (C and D) Migration capacity of RCC cells were examined by wound healing assay. (E and F) Invasion capacity of RCC cells were examined by Matrigel invasion assay. Data were shown as mean ± s.e.m. (n = 3) ***P<0.001.

Pontin mRNA and protein expression was remarkably inhibited in A498, 786-O and KRC/Y cells treated with pontin siRNA1 compared with the control siRNA group (Fig. 4A and B). Then we studied the effects of pontin depletion on cell migration and invasion using would healing assay and Matrigel invasion assay. The potential effect of cell proliferation on cell migration and invasion was rolled out (S1 Fig.). The migratory capacity of pontin siRNA1 treated cells was significantly decreased at 24 h after scratch (the radios of wound closure: control *vs*. pontin siRNA1, A498: 0.626 ± 0.030 *vs*. 0.128 ± 0.022, *P* < 0.0001; 786-O: 0.635 ± 0.020 *vs*. 0.058 ± 0.011, *P* < 0.0001; KRC/Y: 0.440 ± 0.022 *vs*. 0.037 ± 0.013, *P* < 0.0001) (Fig. 4C and D). In addition, Matrigel invasion assay revealed that the number of invaded cells penetrating through the filter was significantly reduced following pontin depletion in A498 (the number of invaded cells per field: control *vs*. pontin siRNA1: 80.67 ± 3.64 *vs*. 15.67 ± 1.74, *P* < 0.0001), 786-O (87.67 ± 9.67 *vs*. 5.00 ± 1.15, *P* < 0.0001) and KRC/Y (77.50 ± 3.18 *vs*. 14.33 ± 1.67, *P* < 0.0001) (Fig. 4E and F). Additionally, we examine the potential off target effects of pontin siRNA1 by introducing a nonoverlapping pontin siRNA2. The results ruled out the nonspecific off target effects of the siRNA approach (S2 Fig.).

### Effects of pontin depletion on the expression of E-cadherin and vimentin, and β-catenin localization

**Fig 5:**
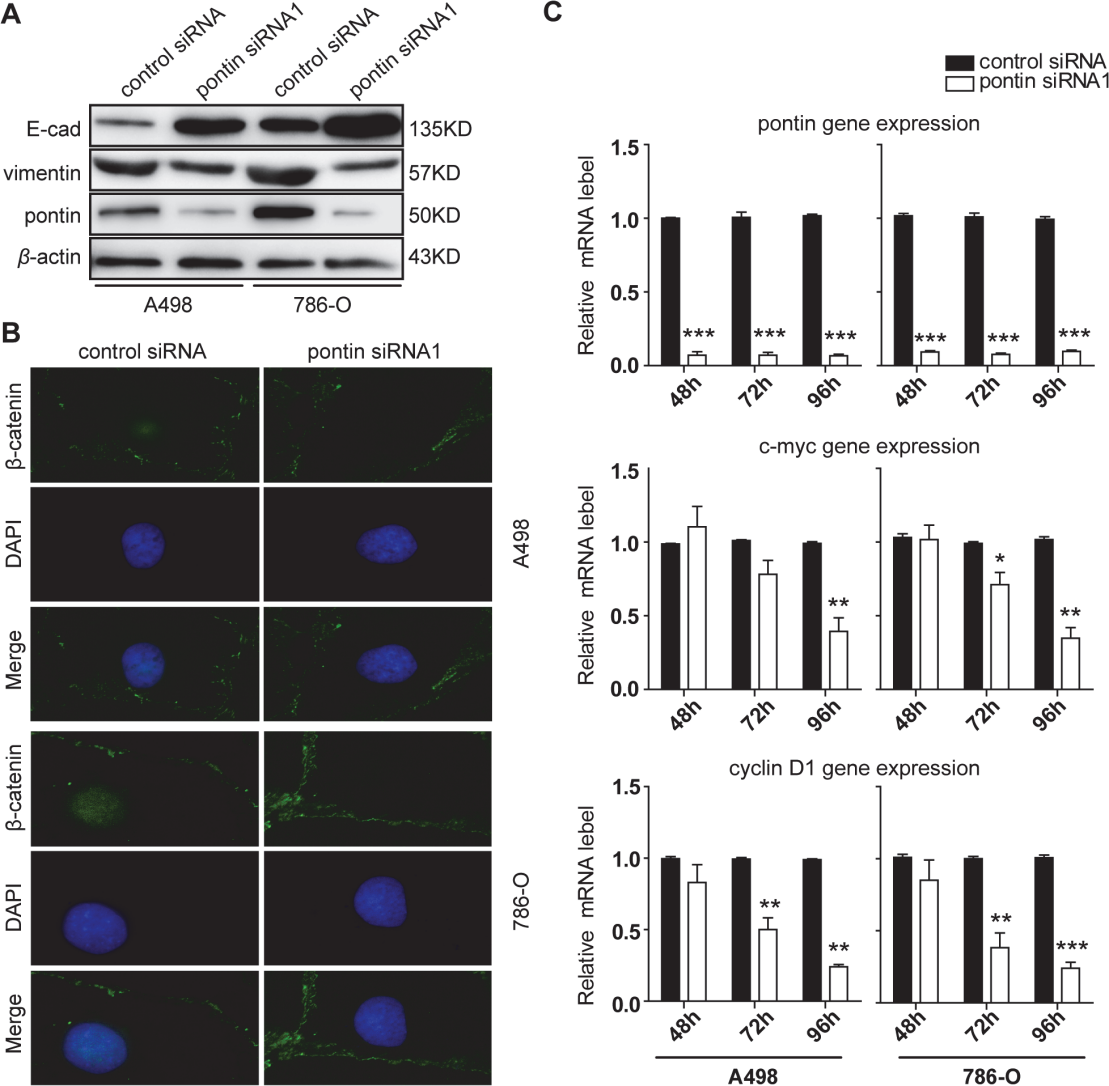
Effects of pontin depletion on the expression of E-cadherin and vimentin, and *β*-catenin subcellular localization *in vitro*. (A) Effects on the protein expression of pontin, E-cadherin and vimentin after siRNA transfection for 72 h detected by western blotting. The data shown is representative of 3 independent experiments. (B) The subcellular location of *β*-catenin was measured by fluorescent immunocytochemical analysis after siRNA transfection for 72 h (green, β-catenin; blue, DAPI nuclear staining). Magnification: ×600. (C) Time dependent changes in mRNA levels of pontin, c-myc and cyclin D1 after siRNA transfection for 48h, 72h and 96h detected by qRT-PCT. Data were shown as mean ± s.e.m. (n = 3). *β*-actin was used as a loading control. *P<0.05, **P<0.01, ***P<0.001.

Previous studies demonstrated that the epithelial-to-mesenchymal transition (EMT) is a crucial step for epithelial cancer progression. We hypothesized that pontin might regulate the metastatic progression of RCC through EMT pathway, and investigate the potential effect of pontin on E-cadherin, vimentin protein expression. The results revealed that E-cadherin was dramatically increased in the pontin siRNA1 treated cells as compared with the control siRNA group, while vimentin was significantly decreased following the pontin depletion (Fig. 5A).

Furthermore, we investigate the potential effect of pontin depletion on *β*-catenin. Fluorescence immunocytochemical analysis revealed that nuclear *β*-catenin was significantly decreased in the pontin siRNA1 treated cells than in the control siRNA group (Fig. 5B). In consistence, qRT-PCR results demonstrated a significant decrease of the mRNA levels of 2 canonical *β*-catenin target gene c-myc [22] and cyclin D1 [23] in pontin siRNA1 treated group in a time-dependent manner, compared with the control siRNA group (Fig. 5C), indicating the potential effect of pontin on *β*-catenin nuclear translocation.

## Discussion

Recent studies have implicated the AAA+ superfamily member pontin and its homology reptin are involved in many cellular processes that are highly relevant to cancer. Both proteins interact with some major oncogenic factors such as *β*-catenin and c-myc, and regulate their oncogenic functions [7, 8]. Several studies revealed aberrant expression of pontin and reptin in some human progressive malignancies: both pontin and reptin are overexpressed in hepatocellular carcinoma [10, 12, 24], pontin overexpression are reported in colorectal [13, 14], prostate [25], and non-small cell lung cancers [26], reptin overexpression are reported in breast [15], gastric [20], and bladder cancers [27]. To the best of our knowledge, there are few studies focused on the role of pontin in RCC, and the molecular mechanism of pontin participating in tumor metastatic progression has so far not been elucidated.

Our study is the first report showing that pontin overexpression widely occurs in kidney cancer. We observed a 2 to 3 fold higher expression of pontin mRNA and protein in tumor tissues than in the matched normal renal tissues in 28 paired clear cell RCC samples. In consistence, IHC staining showed an intense staining of pontin in 91.6% of RCC tissues, especially at the tumorous invasive margin, while only a diffuse pontin staining in proximal tubules was observed in the normal renal tissues. However, the mechanism for the up-regulation of pontin and reptin in cancer remains currently unclear. Pontin gene lies on chromosome 3q21, a region commonly amplified in non-small cell lung cancer, which indicates that pontin overexpression may be due to gene amplification [26]. But that region is not commonly affected in HCC, colorectal cancer, or RCC, thus it is unlikely that pontin up-regulation is consecutive to gene amplification in these cancers. Further study needs to be done to elucidate the molecular mechanisms of pontin overexpression in RCC.

Statistical analysis of the clinicopathological features of RCC patients in our study revealed that high cytoplasmic pontin expression was significantly associated with the aggressive features of RCC. Kim *et al* reported that reptin depletion reduced invasive capacity of the prostatic LNCap cells, which is in accord with the requirement of reptin in the repression of the anti-metastatic gene KAI1 [28]. However, to the best of our knowledge, the effects of pontin on migration and invasion of cancer cells remains unclear, except that Cram *et al* using systemic RNAi screen identified pontin as a member of a cell migration gene network in Caenorhabditis elegans [29]. Our study firstly demonstrated that pontin depletion by siRNA significantly inhibited the migration and invasion capacity of RCC cell lines *in vitro*.

Along our efforts to explore the mechanism of the pro-migratory and pro-invasive role of pontin in RCC, we observed a significantly decreased expression of E-cadherin in ccRCC tissues. Concomitantly, a dramatic increase of E-cadherin expression and a significant decrease of vimentin expression in RCC cell lines following pontin depletion was observed, suggesting the involvement of EMT in the pro-migratory and pro-invasive effect of pontin. For epithelial malignances, EMT is considered to be an essential step in cancer dissemination and metastatic progression [30]. E-cadherin is a marker of the epithelial-like phenotype. Loss of E-cadherin is a hallmark of EMT, which leads to disassembly of adherens junctions, and enhances motility and invasion of tumor cells [31]. *β*-catenin is a component of the adherens junctions. It is known that pontin has an agonistic effect on the transcription of the positive targets of *β*-catenin [8], which is one of the main pathways of pontin involving in carcinogenesis. Whitehead *et al* demonstrated that *β*-catenin phosphorylation induced by mechanical stimulation at tyrosine 654, the site of its interaction with E-cadherin, could increase the nuclear localization of *β*-catenin, up-regulating the transcription of oncogenes Myc and Twist1 [32]. Our results that the nuclear *β*-catenin was significantly decreased following pontin depletion supported the hypothesis that pontin may regulate *β*-catenin by relocating it from the adherens junctions in cytoplasm to the transcriptional complexes in nucleus through EMT process. Our data suggest that siRNA-mediated pontin depletion in RCC cell lines revoke the pontin-induced down-regulation of E-cadherin, and subsequently increased the binding capacity of the adherens junctions for *β*-catenin, leaving less *β*-catenin transferred to the transcriptional complexes in nucleus, inhibiting the transcription of the down-stream oncogenes. Above observations is in accord with the findings by Lauscher *et al* that nuclear co-localization of pontin and *β*-catenin was associated with progression of colorectal carcinomas [14]. However, further studies needs to be done to elucidate the molecular mechanisms involved in the pontin-induced down-regulation of E-cadherin.

Given that all main effects of pontin and reptin are associated with the interactions with multi-protein complexes in nucleus, Lauscher *et al* focused on the nuclear expression of pontin, albeit they found cytoplasmic pontin staining in the majority of colon cancer samples (> 76%) through IHC staining. And they failed to find any correlations between positive nuclear pontin staining and recurrence-free survival or overall survival [13]. In this context, however, one of the highlighted findings is that we first reported the evaluation of subcellular expression of pontin in human RCC, and demonstrated that pontin overexpression in cytoplasm, rather than in nucleus, significantly correlated with RCC metastatic progression. These observations, together with our previous study on reptin [16], strongly indicated that pontin and reptin may have distinct carcinogenic effects localizing in cytoplasm, independent of their known roles in nucleus. Study on HCC also revealed definite cytoplasmic reptin expression in all analyzed HCCs [24], suggesting additional oncogenic properties of reptin in cytoplasm.

In summary, this study for the first time identified pontin up-regulated in RCC and high cytoplasmic pontin expression was associated with poor survival of RCC patients. *In vitro* experiments suggest that pontin may play a previously unknown pro-invasive role in the metastatic progression of RCC through EMT pathway. Therefore, these results warrant further investigation of pontin as a potential therapeutic target in developing more effective treatment of RCC.

## Supporting Information

S1 Fig Effects of pontin depletion on RCC cell proliferation in 24-hour serum-free medium culture. A498 and 786-O cell numbers were counted in 4 randomly selected high-power fields (× 400) per well in the same condition as for migration assay. No significant difference of the cell number between pontin siRNA1 group and the control siRNA group. Data were shown as mean ± s.e.m. (n = 3)
(TIF)

S2 Fig Effects of pontin depletion on RCC cell migration/invasion activities *in vitro*. (A) Effects on the pontin mRNA expression after siRNA transfection for 48 h detected by semi-quantitative RT-PCR. Pontin mRNA was remarkably inhibited in A498 and 786-O treated with pontin siRNA2 compared with the control siRNA group. (B and C) Migration capacity of RCC cells were examined by wound healing assay. The migratory capacity of pontin siRNA2 treated A498 and 786-O was significantly decreased at 24 h after the scratch (both *P* < 0.001). (D and E) Invasion capacity of RCC cells were examined by Matrigel invasion assay. The invasive capacity of pontin siRNA2 treated A498 and 786-O was significantly decreased as compared with the control siRNA group (both *P* < 0.001). Data were shown as mean ± s.e.m. (n = 3) ***P<0.001.
(TIF)

## References

[1] Eisengart LJ, MacVicar GR, Yang XJ. Predictors of response to targeted therapy in renal cell carcinoma. Archives of pathology & laboratory medicine. 2012;136(5):490–5. 10.5858/arpa.2010-0308-RA . 22229848

[2] Grigoletto A, Lestienne P, Rosenbaum J. The multifaceted proteins Reptin and Pontin as major players in cancer. Biochimica et biophysica acta. 2011;1815(2):147–57. 10.1016/j.bbcan.2010.11.002 . 21111787

[3] Jin J, Cai Y, Yao T, Gottschalk AJ, Florens L, Swanson SK, et al A mammalian chromatin remodeling complex with similarities to the yeast INO80 complex. The Journal of biological chemistry. 2005;280(50):41207–12. 10.1074/jbc.M509128200 . 16230350

[4] Gallant P. Control of transcription by Pontin and Reptin. Trends in cell biology. 2007;17(4):187–92. 10.1016/j.tcb.2007.02.005 . 17320397

[5] Jha S, Shibata E, Dutta A. Human Rvb1/Tip49 is required for the histone acetyltransferase activity of Tip60/NuA4 and for the downregulation of phosphorylation on H2AX after DNA damage. Molecular and cellular biology. 2008;28(8):2690–700. 10.1128/MCB.01983-07 18285460 PMC2293106

[6] Boulon S, Marmier-Gourrier N, Pradet-Balade B, Wurth L, Verheggen C, Jady BE, et al The Hsp90 chaperone controls the biogenesis of L7Ae RNPs through conserved machinery. The Journal of cell biology. 2008;180(3):579–95. 10.1083/jcb.200708110 18268104 PMC2234240

[7] Wood MA, McMahon SB, Cole MD. An ATPase/helicase complex is an essential cofactor for oncogenic transformation by c-Myc. Molecular cell. 2000;5(2):321–30. . 10882073 10.1016/s1097-2765(00)80427-x

[8] Bauer A, Chauvet S, Huber O, Usseglio F, Rothbacher U, Aragnol D, et al Pontin52 and reptin52 function as antagonistic regulators of beta-catenin signalling activity. The EMBO journal. 2000;19(22):6121–30. 10.1093/emboj/19.22.6121 11080158 PMC305835

[9] Feng Y, Lee N, Fearon ER. TIP49 regulates beta-catenin-mediated neoplastic transformation and T-cell factor target gene induction via effects on chromatin remodeling. Cancer research. 2003;63(24):8726–34. . 14695187

[10] Haurie V, Menard L, Nicou A, Touriol C, Metzler P, Fernandez J, et al Adenosine triphosphatase pontin is overexpressed in hepatocellular carcinoma and coregulated with reptin through a new posttranslational mechanism. Hepatology. 2009;50(6):1871–83. 10.1002/hep.23215 19877184 PMC2927003

[11] Menard L, Taras D, Grigoletto A, Haurie V, Nicou A, Dugot-Senant N, et al In vivo silencing of Reptin blocks the progression of human hepatocellular carcinoma in xenografts and is associated with replicative senescence. Journal of hepatology. 2010;52(5):681–9. 10.1016/j.jhep.2009.12.029 . 20346530

[12] Berasain C. New therapeutic targets in HCC: reptin ATPase and HCC senescence. Journal of hepatology. 2010;52(5):633–4. 10.1016/j.jhep.2010.01.020 . 20334944

[13] Lauscher JC, Elezkurtaj S, Dullat S, Lipka S, Grone J, Buhr HJ, et al Increased Pontin expression is a potential predictor for outcome in sporadic colorectal carcinoma. Oncology reports. 2012;28(5):1619–24. 10.3892/or.2012.1968 . 22895545

[14] Lauscher JC, Loddenkemper C, Kosel L, Grone J, Buhr HJ, Huber O. Increased pontin expression in human colorectal cancer tissue. Human pathology. 2007;38(7):978–85. 10.1016/j.humpath.2007.01.005 . 17442372

[15] Maslon MM, Hrstka R, Vojtesek B, Hupp TR. A divergent substrate-binding loop within the pro-oncogenic protein anterior gradient-2 forms a docking site for Reptin. Journal of molecular biology. 2010;404(3):418–38. 10.1016/j.jmb.2010.09.035 . 20888340

[16] Ren J, Li W, Liu H, Yan L, Jiao W, Li D, et al Overexpression of reptin in renal cell carcinoma contributes to tumor malignancies and its inhibition triggers senescence of cancer cells. Urologic oncology. 2013;31(7):1358–66. 10.1016/j.urolonc.2012.01.004 . 22341977

[17] Eble J, Sauter G, Epstein J, Sesterhenn Ie. World Health Organization Classification of Tumors Pathology and Genetics of the Urinary Systemandmale Genital Organs. LARC Press: Lyon, pp12–14.; 2004.

[18] Fuhrman SA, Lasky LC, Limas C. Prognostic significance of morphologic parameters in renal cell carcinoma. The American journal of surgical pathology. 1982;6(7):655–63. . 7180965 10.1097/00000478-198210000-00007

[19] Wang F, Grigorieva EV, Li J, Senchenko VN, Pavlova TV, Anedchenko EA, et al HYAL1 and HYAL2 inhibit tumour growth in vivo but not in vitro. PloS one. 2008;3(8):e3031 10.1371/journal.pone.0003031 18725949 PMC2516603

[20] Li W, Zeng J, Li Q, Zhao L, Liu T, Bjorkholm M, et al Reptin is required for the transcription of telomerase reverse transcriptase and over-expressed in gastric cancer. Molecular cancer. 2010;9:132 10.1186/1476-4598-9-132 20509972 PMC2887797

[21] Wolf D, Wolf AM, Rumpold H, Fiegl H, Zeimet AG, Muller-Holzner E, et al The expression of the regulatory T cell-specific forkhead box transcription factor FoxP3 is associated with poor prognosis in ovarian cancer. Clinical cancer research: an official journal of the American Association for Cancer Research. 2005;11(23):8326–31. 10.1158/1078-0432.CCR-05-1244 . 16322292

[22] He TC, Sparks AB, Rago C, Hermeking H, Zawel L, da Costa LT, et al Identification of c-MYC as a target of the APC pathway. Science. 1998;281(5382):1509–12. . 9727977 10.1126/science.281.5382.1509

[23] Tetsu O, McCormick F. Beta-catenin regulates expression of cyclin D1 in colon carcinoma cells. Nature. 1999;398(6726):422–6. 10.1038/18884 . 10201372

[24] Rousseau B, Menard L, Haurie V, Taras D, Blanc JF, Moreau-Gaudry F, et al Overexpression and role of the ATPase and putative DNA helicase RuvB-like 2 in human hepatocellular carcinoma. Hepatology. 2007;46(4):1108–18. 10.1002/hep.21770 . 17657734

[25] Kim JH, Lee JM, Nam HJ, Choi HJ, Yang JW, Lee JS, et al SUMOylation of pontin chromatin-remodeling complex reveals a signal integration code in prostate cancer cells. Proceedings of the National Academy of Sciences of the United States of America. 2007;104(52):20793–8. 10.1073/pnas.0710343105 18087039 PMC2410081

[26] Dehan E, Ben-Dor A, Liao W, Lipson D, Frimer H, Rienstein S, et al Chromosomal aberrations and gene expression profiles in non-small cell lung cancer. Lung cancer. 2007;56(2):175–84. 10.1016/j.lungcan.2006.12.010 . 17258348

[27] Sanchez-Carbayo M, Socci ND, Lozano J, Saint F, Cordon-Cardo C. Defining molecular profiles of poor outcome in patients with invasive bladder cancer using oligonucleotide microarrays. Journal of clinical oncology: official journal of the American Society of Clinical Oncology. 2006;24(5):778–89. 10.1200/JCO.2005.03.2375 . 16432078

[28] Kim JH, Kim B, Cai L, Choi HJ, Ohgi KA, Tran C, et al Transcriptional regulation of a metastasis suppressor gene by Tip60 and beta-catenin complexes. Nature. 2005;434(7035):921–6. 10.1038/nature03452 . 15829968

[29] Cram EJ, Shang H, Schwarzbauer JE. A systematic RNA interference screen reveals a cell migration gene network in C. elegans. Journal of cell science. 2006;119(Pt 23):4811–8. 10.1242/jcs.03274 . 17090602

[30] Thiery JP, Acloque H, Huang RY, Nieto MA. Epithelial-mesenchymal transitions in development and disease. Cell. 2009;139(5):871–90. 10.1016/j.cell.2009.11.007 . 19945376

[31] Baum B, Georgiou M. Dynamics of adherens junctions in epithelial establishment, maintenance, and remodeling. The Journal of cell biology. 2011;192(6):907–17. 10.1083/jcb.201009141 21422226 PMC3063136

[32] Whitehead J, Vignjevic D, Futterer C, Beaurepaire E, Robine S, Farge E. Mechanical factors activate beta-catenin-dependent oncogene expression in APC mouse colon. HFSP journal. 2008;2(5):286–94. 10.2976/1.2955566 19404440 PMC2639941

