# Supplementary figures and images for: Cytoplasmic Expression of Pontin in Renal Cell Carcinoma Correlates with Tumor Invasion, Metastasis and Patients’ Survival
Source: PLoS One. 2015 Mar 9;10(3):e0118659. doi: 10.1371/journal.pone.0118659 (PMC4353622; doi:10.1371/journal.pone.0118659)

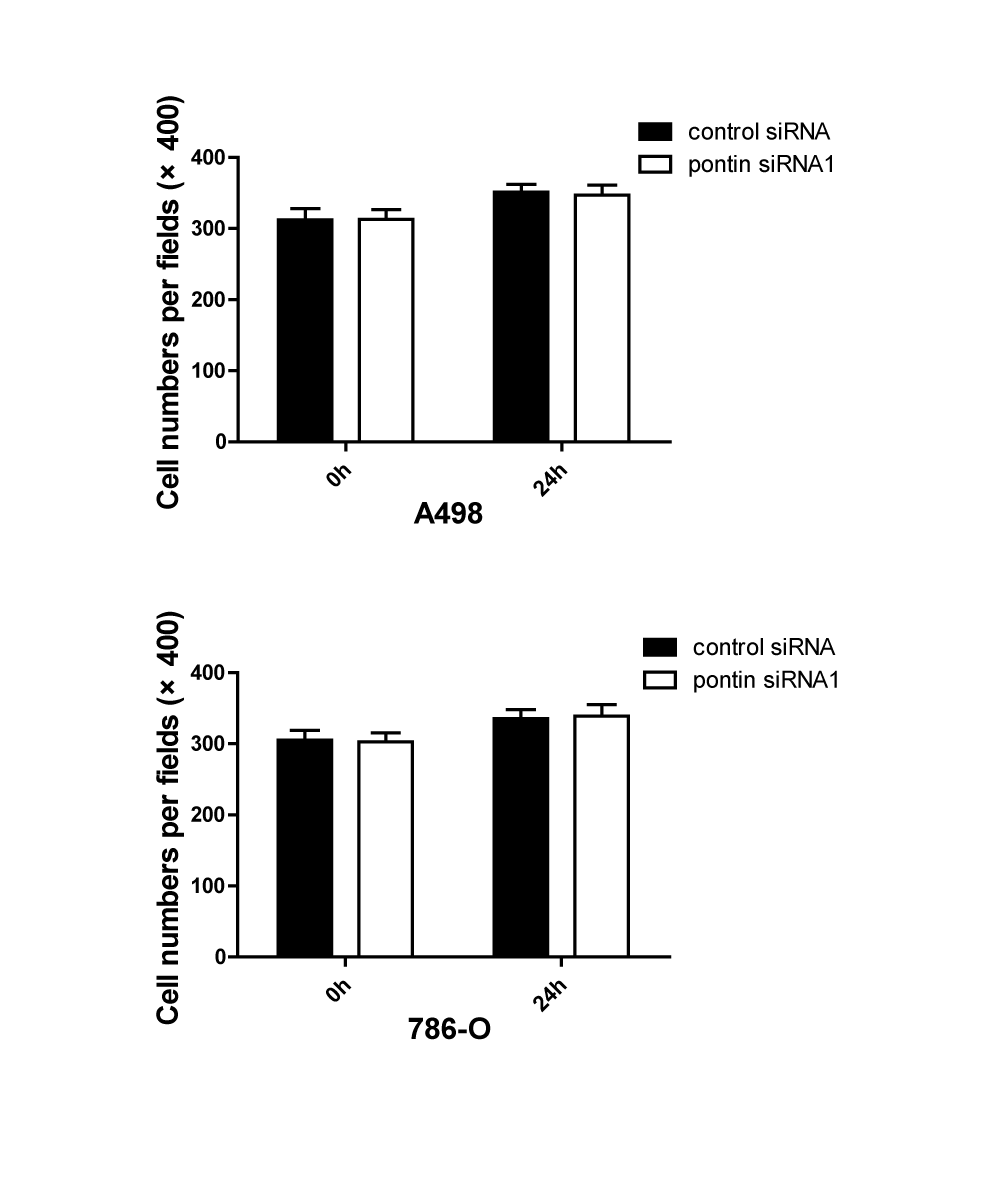

Supplement: S1 Fig — A498 and 786-O cell numbers were counted in 4 randomly selected high-power fields (× 400) per well in the same condition as for migration assay. No significant difference of the cell number between pontin siRNA1 group and the control siRNA group. Data were shown as mean ± s.e.m. (n = 3) (TIF) [file pone.0118659.s001.tif]

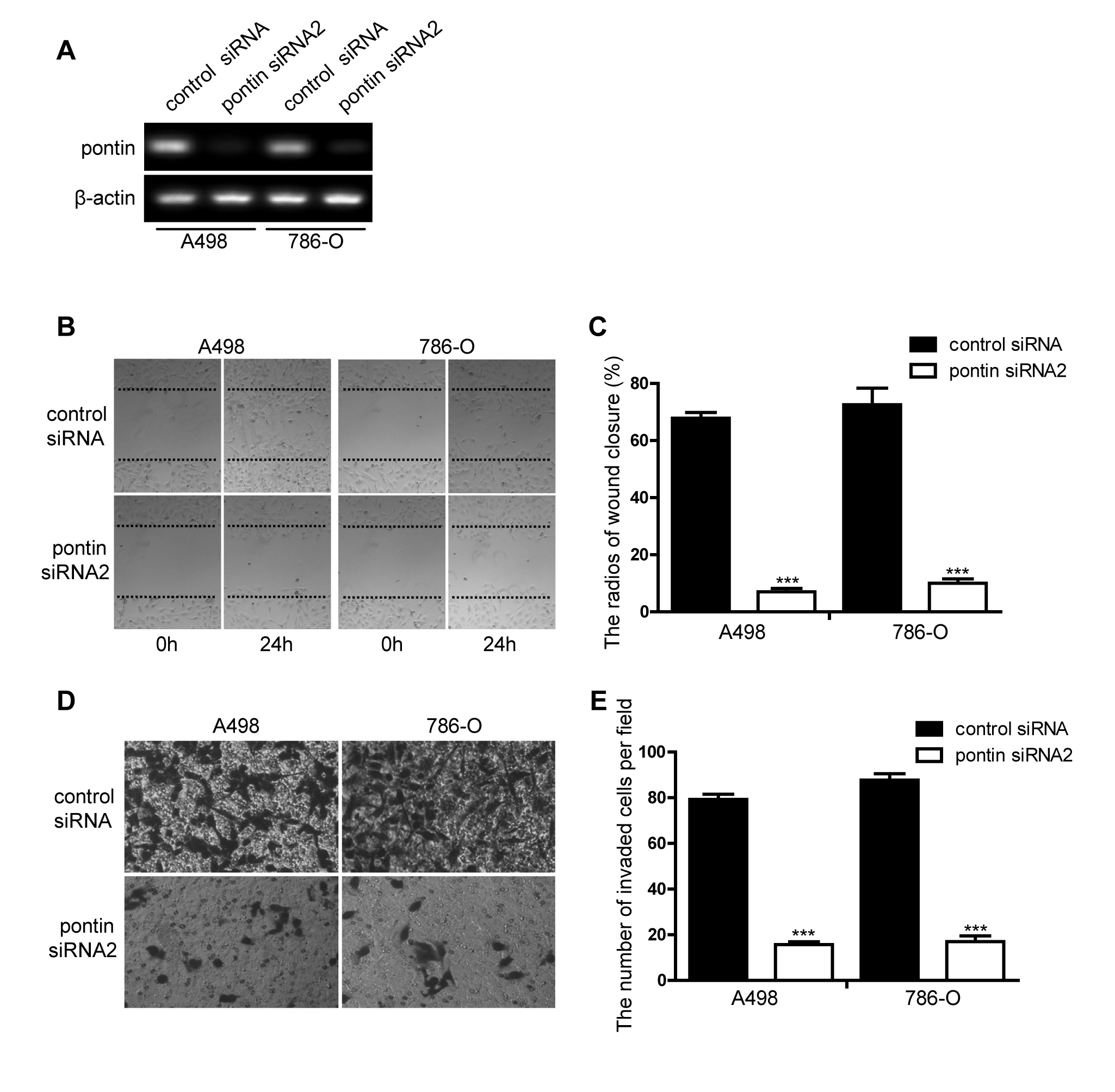

Supplement: S2 Fig — (A) Effects on the pontin mRNA expression after siRNA transfection for 48 h detected by semi-quantitative RT-PCR. Pontin mRNA was remarkably inhibited in A498 and 786-O treated with pontin siRNA2 compared with the control siRNA group. (B and C) Migration capacity of RCC cells were examined by wound healing assay. The migratory capacity of pontin siRNA2 treated A498 and 786-O was significantly decreased at 24 h after the scratch (both P < 0.001). (D and E) Invasion capacity of RCC cells were examined by Matrigel invasion assay. The invasive capacity of pontin siRNA2 treated A498 and 786-O was significantly decreased as compared with the control siRNA group (both P < 0.001). Data were shown as mean ± s.e.m. (n = 3) ***P<0.001. (TIF) [file pone.0118659.s002.tif]
